# T-DNA Mutagenesis Reveals FpPer1 as a Dual-Function Regulator of Virulence and Fungicide Resistance in *Fusarium pseudograminearum*

**DOI:** 10.3390/jof11090673

**Published:** 2025-09-12

**Authors:** Haiyang Li, Panpan Zhang, Xueqian Song, Huiying Li, Cong Chen, Limin Wang, Zhifang Wang, Lingjun Hao, Yun Li, Xinlong Wang, Jiangang Kang, Honglian Li, Min Wang, Shengli Ding

**Affiliations:** College of Plant Protection, Henan Agricultural University, Zhengzhou 450046, China

**Keywords:** *Fusarium* crown rot, *Fusarium pseudograminearum*, *FpPER1*, pathogenicity, fungicide

## Abstract

*Fusarium* crown rot (FCR), caused by *Fusarium pseudograminearum*, is a devastating wheat disease leading to significant yield losses worldwide. However, the pathogenic mechanism of *F. pseudograminearum* and its resistance to fungicides remain poorly understood. In this study, we identified a hypothetical gene encoding GPI-anchored protein, designated FpPer1, by screening a T-DNA insertion mutant library of *F. pseudograminearum* for tebuconazole resistance. The Δ*Fpper1* mutant exhibited increased sensitivity to the triazole antifungal drugs and fludioxonil. Additionally, the deletion of *FpPER1* impaired fungal growth, conidiation, and pathogenicity in barley leaves and wheat coleoptiles. Furthermore, the Δ*Fpper1* mutant displayed enhanced susceptibility to various environmental stresses, including NaCl, CR, sorbitol, H_2_O_2_, and SDS. The mutant also showed reduced penetration peg formation and impaired reactive oxygen species (ROS) scavenging ability during infection. Subcellular localization analysis revealed that FpPer1-GFP co-localized with the endoplasmic reticulum (ER) marker RFP-HDEL in both conidia and hyphae, indicating its localization in the ER. In summary, our findings demonstrate that *FpPER1* plays an important role in pathogenicity and fungicide resistance in *F. pseudograminearum*. This study not only provides a theoretical foundation for understanding fungal virulence mechanisms but also offers practical insights for developing novel fungicide strategies.

## 1. Introduction

Wheat is one of the most important crops globally, with production steadily increasing due to advances in breeding techniques and agronomic management [1,2]. However, several factors hinder wheat production. *Fusarium* crown rot (FCR) caused by *F. pseudograminearum* is a soil-borne wheat disease, firstly reported in China by our research group [3]. *F. pseudograminearum* can infect wheat throughout its entire growth cycle, causing FCR through coleoptile and root infection or *Fusarium* head blight (FHB) via spike colonization, both of which significantly compromise grain yield and quality [4,5,6]. Recently, the lack of available resistant varieties [7], coupled with limited effective control measures, has led to an increase in FCR incidence, impacting approximately 3.3 million hectares in China’s Huanghuai wheat area in 2024. Therefore, exploring the growth and development mechanisms, and pesticide resistance of *F. pseudograminearum* is crucial for understanding the disease’s occurrence and provides a theoretical basis for developing effective prevention and control strategies.

As FCR becomes a serious disease in wheat production, researchers are increasingly focusing on the molecular mechanisms that regulate the biological function in *F. pseudograminearum*. Recent studies have made significant progress in identifying key regulatory genes that influence pathogenicity in *F. peseudograminearum*. The key component of the Rpd3S complex, FpRco1, has been characterized to modulate vegetative growth and pathogenicity in *F. pseudograminearum* through mutant library screening [8]. Liu reported that FpZRA1 plays an important role in regulating fungal development, deoxynivalenol (DON) biosynthesis, and pathogenicity, with its expression induced by fungicides [9]. The transcription factor Fp487 is essential for growth and virulence in *F. pseudograminearum*, making it a potential RNAi target for RNA interference (RNAi) in managing FCR [10]. In addition, recent advances in pathogen-host interaction studies have identified and characterized two pathogen-associated molecular patterns (PAMPs), namely FpCDP1 and Fp00392 [11,12]. In summary, several key genes involved in the growth and pathogenicity of *F. pseudograminearum* have already been identified.

Targeting key genes that regulate fungal growth, development, and pathogenicity has emerged as a pivotal strategy for fungicide exploitation. Benzimidazole fungicides target fungal β-tubulin, effectively inhibiting microtubule assembly and disrupting essential cellular processes [13]. Similarly, phenamacril specifically binds to myosin I (MyoI), inhibiting its ATPase activity [14]. Sterol demethylation inhibitors (DMIs) block the demethylation process of ergosterol, leading to fungal cell death [15]. Succinic dehydrogenase inhibitors (SDHIs) block the activity of succinate dehydrogenase, ultimately disrupting fungal growth [16]. However, emerging studies have demonstrated that the long-term use of pesticides can lead to the development of fungicide resistance [17]. Ipconazole is an effective fungicide against *F. pseudograminearum*; however, the point mutation G464S of FpCYP51B has been found to confer resistance to ipconazole in the pathogen [18]. Therefore, identifying novel pesticide targets and understanding their resistance mechanisms are crucial for the development of new fungicide.

Glycosylphosphatidylinositol (GPI) is a posttranslational modification that plays an important role in anchoring cell-surface proteins to membranes via lipid modification in eukaryotes [19]. Numerous studies have indicated that GPI-anchored proteins are implicated in a wide range of cellular processes. It was found that the GPI-anchored protein ScPer1 was required for GPI-phospholipase A2 activity, and the mutant Δscper1 was functionally complemented by human ortholog PERLD1 [20]. In plant fungal pathogen *Cochlibolus heterostrophus*, the GPI ethanolamine phosphate transferase gene *ChGPI7* is critical for infection by regulating cell wall integrity and immune evasion [21]. These findings highlight the pivotal roles of GPI-anchored proteins across various biological functions.

Although some functions of the GPI-anchored proteins in pathogens have been elucidated, many issues remain unresolved. In this study, we employed a forward genetics approach to identify the genes targeted by fungicide via screening T-DNA insertion mutants. Our results determined that FpPer1 plays a crucial role in growth and pathogenicity, particularly in enhancing tolerance to fungicides, which may aid in the development of new pesticides in the future.

## 2. Materials and Methods

### 2.1. Strain and Culture Conditions

The wild-type *F. pseudograminearum* (WZ-8A), *Escherichia coli* (DH5α), and vectors pKOV21 containing the hygromycin phosphotransferase gene (*HPH*), pKNTG containing the green fluorescent protein gene (*GFP*), and pKOV21-*HDEL*-*RFP* were used in this study. The following culture media were employed: PDA medium was used for the general culture of *F. pseudograminerum*; YEPD medium for mycelium collecting; Carboxymethyl cellulose (CMC) for spore collection of *F. pseudograminearum*; and LB medium was used for bacterial culture. All required primers were synthesized by Shanghai Bioengineering with some sequences presented in Table S1.

### 2.2. Knockout and Complementation of the FpPER1 Gene

The candidate gene *FpPER1* was deleted in *F. pseudograminearum* using split-marker methods. Initially, approximately 1.0 kb of the fragments of upstream L1 and downstream L2 were amplified using primers 1F/2R and 3F/4R, respectively, with the genome of WZ-8A strain serving as the template. Subsequently, using the fusion fragments L1, *HPH*, and L2 as templates, the fragments of L1H1 and L2H2 were amplified via primers 1F/HY-R and HY-F/4R, respectively. Finally, the mixed fragments of L1H1 and L2H2 were employed for PEG-mediated transformation to knock out the targeting gene (*FpPER1*) in WZ-8A. The primers utilized were shown in Table S1.

We conducted the complementation experiment on the mutant following the knockout experiment adhering to the aforementioned PEG-mediated protoplast transformation method. The *FpPER1* gene with its native promoter was cloned into the pKNTG vector. The complementation transformations of c*FpPer1* were screened using G418 resistance and confirmed by PCR with primers of NF/NR.

### 2.3. Biological Assays

The fungal growth rate was measured firstly, and the deletion mutant, complementation mutants, and wild-type (WZ-8A) were inoculated on PDA plates for 3 d at 25 °C. Subsequently, the diameters of the different strains were measured. For conidiation assays, fresh mycelial blocks were inoculated in CMC medium with shaking at 150 rpm for 5 d at 25 °C. Those experiments were performed in triplicate with three treatments each.

### 2.4. Pathogenicity Assay

Barley leaves, cultivated for 5 days, were used for inoculation with 5 mm fungal blocks. The fungal blocks were removed after 24 h of dark incubation. The plants were photographed after 3 d. Alternatively, when coleoptiles grew to 2–3 cm in length, wounds at the stem base were inoculated with 2 μL of conidial suspension at a concentration of 1.0 × 10^7^/mL in ddH_2_O after the apex was cut. The treatments were maintained at 25 °C in a moist chamber in the dark for 24 h, and then the plants were subsequently photographed after being cultured under light/night for one week.

### 2.5. Subcellular Localization of FpPer1

The wild-type strain of *F. pseudograminearum* was co-transformed with the pKNTG-*FpPER1* plasmid and the pKOV21-*RFP*-*HDEL* plasmid using the previously mentioned PEG-mediated method. The hyphae of the co-transformed strains were cultured and collected, and fluorescence localization of FpPer1 was observed in the co-transformed strains.

### 2.6. Sensitivity Assays to Different Fungicides

The technical-grade active ingredients of tebuconazole, difenoconazole, epoxiconazole, and fludioxonil were separately dissolved in 1 mL of acetone and then diluted with sterile water to prepare 5 ppm solutions. Under sterile conditions, an appropriate amount of the prepared pesticide solution was added to sterilize PDA medium in conical flasks to prepare PDA plates containing the corresponding fungicide concentrations (0.084 ppm Tebuconazole, 0.4 ppm Difenoconazole, 0.1 ppm Epoxiconazole, and 0.05 ppm Fludioxonil), followed by thorough mixing. Then, 15 mL of the mixture was poured into 9 cm-diameter Petri dishes. Fresh mycelial plugs were inoculated onto the fungicide-containing plates and control plates for cultivation, and the inhibitory effects of the respective fungicides were calculated.

## 3. Results

### 3.1. Identification of Tebuconazole Resistance-Related Genes by Screening Mutant Library

**Figure 1:**
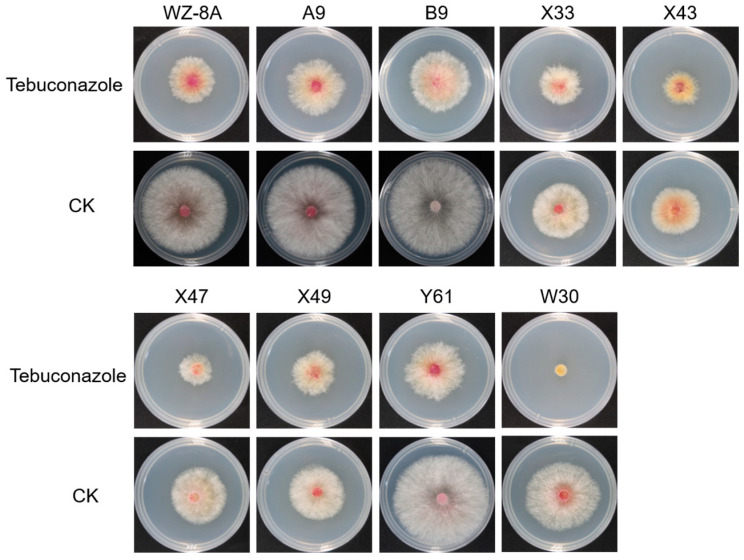
Identification of T-DNA insertion mutants associated with tebuconazole resistance. The top panel displays the mycelial growth of *F. pseudograminearum* on PDA plates supplemented with tebuconazole, while the bottom panels show the mycelial growth on PDA plates without tebuconazole (control). The colonies were measured and photographed two days post-inoculation on PDA plates. All experiments were conducted at least three times with independent biological replicates.

**Figure 2:**
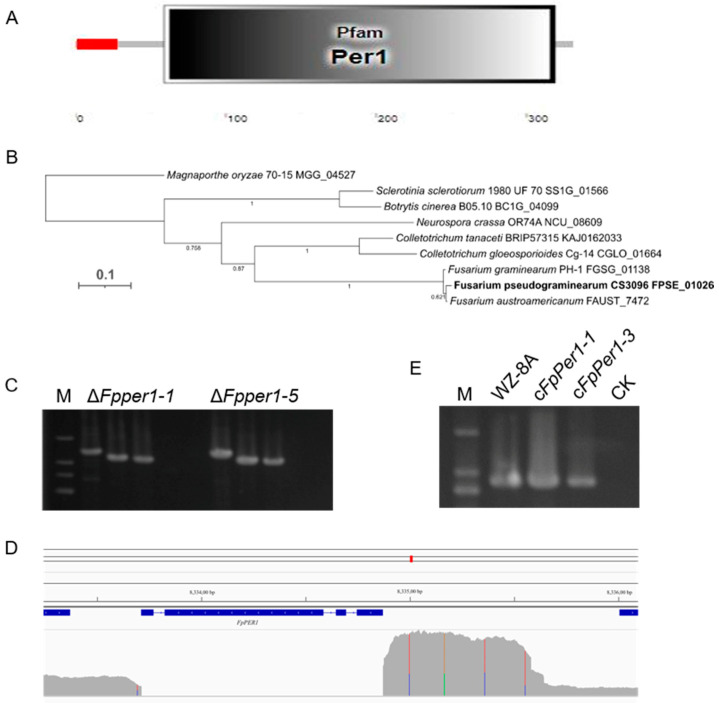
Deletion and complementation of *FpPER1* in *F. pseudograminearum*. (**A**), structural domain of FpPer1, predicted using Smart (http://smart.embl.de/) (accessed on 1 May 2019). The red square indicates signal peptide domains that direct protein transportation, while the gray square represents the Per1-like domain, a GPI-anchoring structure. (**B**), the phylogenetic evolutionary tree of FpPer1 (the bold text) and its orthologs, constructed using MrBayes v 3.1.2. (**C**), the agarose gel results of the PCR screening, revealing the products of in sizes of 1385 bp, 1053 bp, 971 bp, and 861 bp for the Δ*Fpper1-1* and Δ*Fpper1-5* mutants, using four pairs of primers: HYG-F/HYG-R, 1F/2R, 3F/4R, and NF/NR, respectively. (**D**), confirming the complete deletion of *FpPER1* in the genome of the Δ*Fpper1-1* mutant through genomic resequencing, which was performed using the WZ-8A genome as the reference. (**E**), the PCR screening products for WZ-8A, c*FpPer1-1* and c*FpPer1-3* obtained with primer NF/NR, resulting in a product size in 861 bp.

Tebuconazole represents one of the most effective fungicides for controlling FCR. However, its precise mode of action against *F. pseudograminearum* remains incompletely understood. To investigate this, we screened our previously established T-DNA insertion mutant library [8] and identified significant variations in tebuconazole sensitivity among different mutants compared to the wild-type WZ-8A strain. Among them, the mutant W30 exhibited a markedly decreased resistance to tebuconazole (Figure 1). The T-DNA insertion position was confirmed within the FPSE_01026 gene in *F. pseudograminearum* via nested-PCR (Figure S1). The gene encodes the predicted protein with 331 amino acids. Functional domain analysis of the protein encoded by the FPSE_01026 gene revealed that this protein contains signal peptide in N terminal and Per1-like domain in C terminal (Figure 2A), consequently designating it as FpPer1. Phylogenetic analysis has uncovered that FpPer1 in *F. pseudograminearum* exhibits a relatively high affinity with *Fusarium austroamericanum* and *Fusarium graminearum*, compared to *Magnaporthe oryzae*, *Neurospora crassa*, *Colletotrichum tanaceti*, *Colletotrichum gloeosporioides*, *Sclerotinia sclerotiorum*, and *Botrytis cinerea* (Figure 2B).

### 3.2. FpPER1 Is Involved in Growth and Conidia Production of F. pseudograminearum

**Figure 3:**
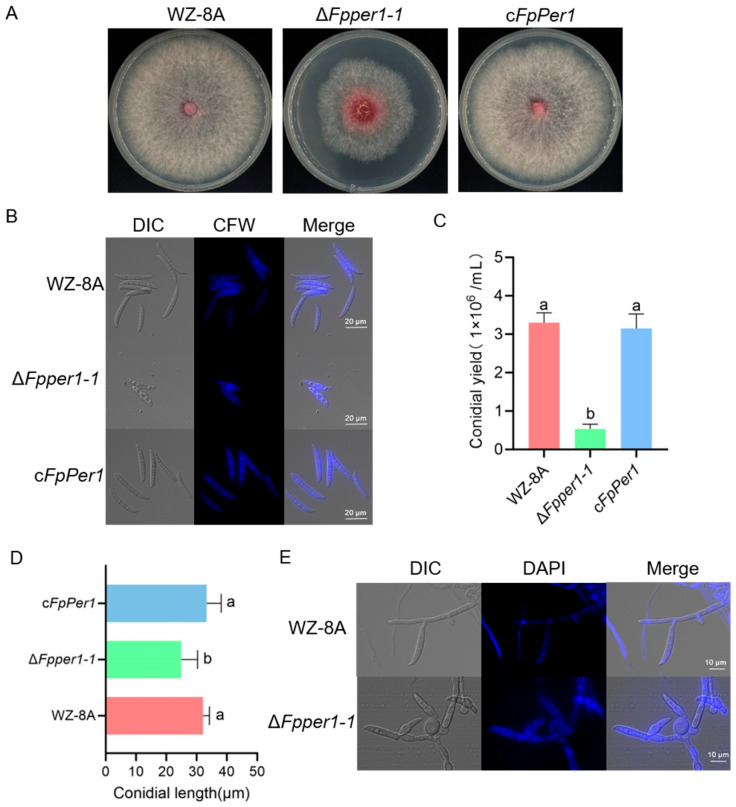
FpPer1 regulates the vegetative growth of *F. pseudograminearum*. (**A**), comparison of colony morphology among the strains WZ-8A, Δ*Fpper1-1*, and the c*FpPer1* on PDA plates. (**B**–**E**), *FpPER1* is involved in conidial formation in *F. pseudograminearum*. (**B**), WZ-8A, Δ*Fpper1-1*, and the complementary strain were cultured in CMC medium for 5 days, photographed using a microscope, and conidia were stained by calcofluor white (CFW) (scale bars = 20 µm). (**C**), the number of conidia was counted. (**D**), the length of conidia was measured. (**E**), the morphology of the mycelium was observed after culturing in CMC medium (scale bars = 10 µm). The mean values ± SD are presented, with lowercase letters (a, b) indicating groups of significant differences as determined by Duncan’s multiple comparison procedures (*p* < 0.05). All experiments were conducted at least three times.

To elucidate the function of FpPer1 in fungicides resistance and other biological processes, we generated *FpPER1* knockout using the split-marker method. Among the transformants obtained, the deletion of gene *FpPER1* in two transformants was verified by PCR (Figure 2C). Furthermore, the whole-genome resequencing of Δ*Fpper1-1* confirmed the complete deletion of the *FpPER1* locus form the *F. pseudograminearum* genome (Figure 2D). Additionally, we constructed complementary strain (c*FpPer1*) that reintroduced *FpPER1* under its native promoter (Figure 2E). Phenotypic characterization revealed that the Δ*Fpper1* mutant exhibited a significantly reduced growth rate and impaired aerial hyphae formation compared to both WZ-8A and complemented strains, indicating the critical role of *FpPER1* in vegetative growth (Figure 3A). Furthermore, the mutant displayed severe conidiation defects, including a reduction in conidial production and abnormal conidial morphology characterized by smaller size. Microscopic analysis revealed that the conidial septum decreased (Figure 3B–D). Regarding the decrease in sporulation, we observed that Δ*Fpper1* exhibited significant hyphal expansion in CMC medium (Figure 3E). Through observation and analysis of sporulation, we determined that the *FpPER1* gene significantly influences the sporulation of *F. pseudograminearum*. In other words, the *FpPER1* gene impacts the reproduction of *F. pseudograminearum*.

### 3.3. Deletion Mutant ΔFpper1 Shows Increased Sensitivity to Various Stress Conditions

**Figure 4:**
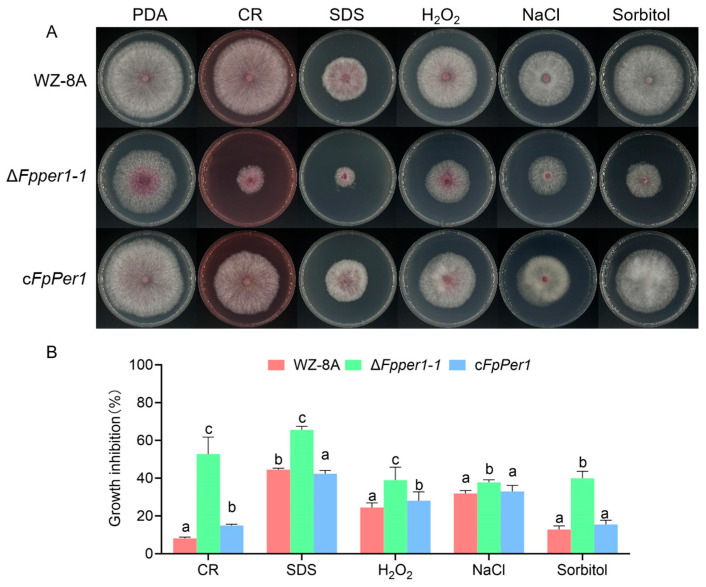
Sensitivity assays of the WZ-8A, Δ*Fpper1-1*, and c*FpPer1* strains under various stress conditions. (**A**), colonies of the strains on PDA medium, both without and with 0. 2 mg/mL Congo Red (CR), 0.025% SDS, 10 mM H_2_O_2_, 1 M NaCl, and 1.5 M Sorbitol. (**B**), colony diameters were measured, with the inhibition rates calculated under different stress conditions. The mean values ± SD from three repetitions are shown, with lowercase letters (a–c) indicating significantly different groups as calculated by Duncan’s multiple comparison procedures (*p* < 0.05).

During pathogen infection of the host, it encounters various environmental stresses, including oxidation, osmotic, and cell wall integrity stresses. To investigate the response of WZ-8A, Δ*Fpper1*, and the complementary strain under different stress conditions, these strains were cultured on PDA supplemented with NaCl, Congo Red (CR), sorbitol, H_2_O_2_, and SDS. Colony diameters were measured, and inhibition rates under different stress conditions were calculated. The results indicated that the deletion of *FpPER1* resulted in increased sensitivity to NaCl, CR, sorbitol, H_2_O_2_, and SDS compared to WZ-8A and complemented strains (Figure 4A,B). These findings demonstrated that *FpPER1* plays an important role in *F. pseudograminearum*’s adaptation to diverse environmental stresses.

### 3.4. FpPER1 Is Essential for Full Virulence in F. pseudograminearum

**Figure 5:**
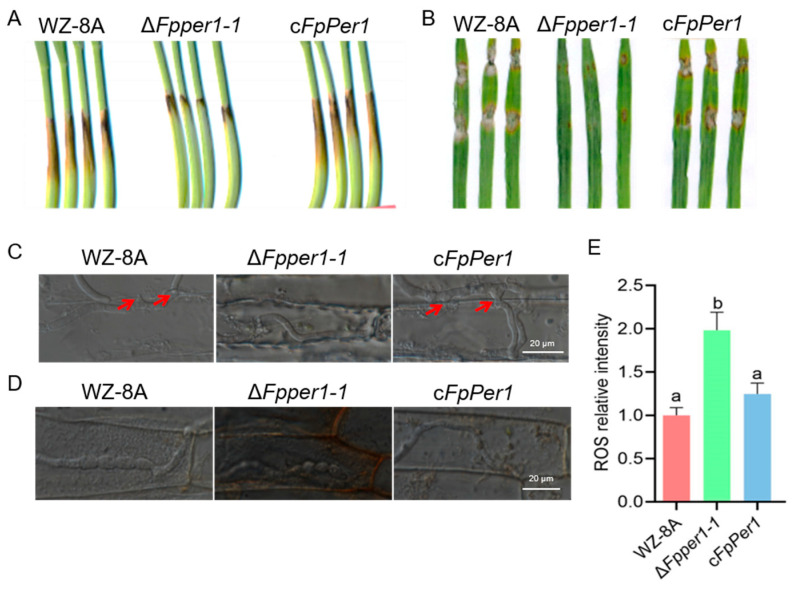
Pathogenicity assay of WZ-8A, Δ*Fpper1-1*, and c*FpPer1*. (**A**), the pathogenicity of these strains in coleoptiles inoculated with conidial suspension for 7 days. (**B**), the pathogenicity of the different strains in barley leaves with inoculated fresh fungal blocks for 3 days. (**C**), the infection process of the different strains in wheat coleoptile cell was observed under a microscope (scale bars = 20 µm), and the red arrow indicates the location of penetration peg formation. (**D**), ROS accumulation in infected coleoptiles was analyzed through DAB staining (scale bars = 20 µm). (**E**), the relative levels of reactive oxygen species (ROS) were measured in cells following infection with different strains. The mean values ± SD are presented, with letters (a, b) indicating groups of significant differences as determined by Duncan’s multiple comparison procedures (*p* < 0.05). All experiments were performed in triplicate.

To assess the pathogenicity of Δ*Fpper1*, conidial suspensions of WZ-8A, the Δ*Fpper1* mutant, and the complementary strain were inoculated onto the wounded wheat coleoptiles. The lesions caused by the Δ*Fpper1* mutant were significantly smaller than those caused by WZ-8A and complemented strain (Figure 5A). Similarly, pathogenic assays on barley leaves revealed that the Δ*Fpper1* mutant exhibited reduced virulence compared to the WZ-8A and complemented strains (Figure 5B). To investigate the underlying cause of this reduced pathogenicity of Δ*Fpper1*, we examined the infection process in wheat coleoptiles. Microscopic observations demonstrated that WZ-8A and complemented strains efficiently formed penetration pegs to facilitate host invasion, whereas Δ*Fpper1* exhibited a marked reduction in penetration peg formation (Figure 5C). Given that Δ*Fpper1* showed increased sensitivity to H_2_O_2_ in vitro, we further evaluated whether FpPer1 is important for scavenging reactive oxygen species (ROSs) during early infection. DAB staining revealed that stronger brown discoloration in Δ*Fpper1*-infected coleoptiles cells compared to those infected by the WZ-8A and complementary strain, suggesting higher ROS accumulation in the absence of *FpPER1* (Figure 5D,E). These results indicate that *FpPER1* regulates penetration peg formation, thereby affecting the pathogenicity of *F. pseudograminearum*, and also modulates host immunity by mitigating ROS accumulation.

### 3.5. FpPer1 Locates in the Endoplasmic Reticulum in F. pseudograminearum

**Figure 6:**
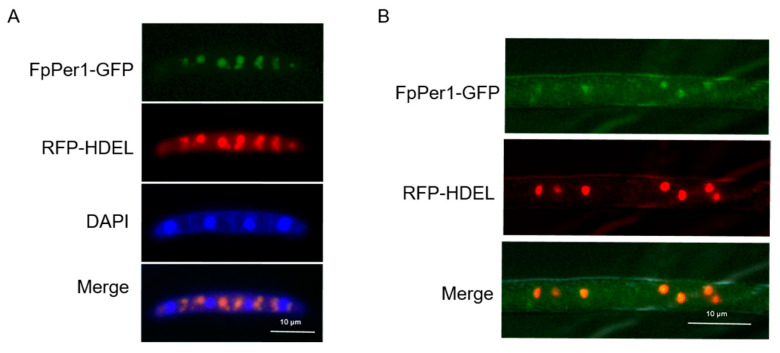
Subcellular localization of FpPer1 in *F. pseudograminearum*. (**A**), the fluorescence of FpPer1-GFP and RFP-HDEL in conidia, observed using a fluorescence microscope, with DAPI employed for nuclear staining (scale bars = 10 µm). (**B**), the fluorescence of FpPer1-GFP and RFP-HDEL in the hyphae of *F. pseudograminearum* was observed (scale bars = 10 µm). All experiments were conducted three times.

Previous studies have reported that the Per1 protein is typically located in the endoplasmic reticulum (ER), which is required for GPI-phospholipase A2 activity [20]. To explore the subcellular localization of FpPer1 in *F. pseudograminearum*, we co-transformed two vectors, encoding FpPer1-GFP and the ER marker RFP-HDEL into the wild-type strain. The results showed that the green fluorescent signal and red fluorescent signal completely merged in conidia, but did not merge with the blue signal, indicating that FpPer1 is localized in the ER, not the nucleus (Figure 6A). Furthermore, we observed identical ER localization patterns for FpPer1 during the hyphae stage (Figure 6B). These results demonstrate that FpPer1 functions in the endoplasmic reticulum of *F. pseudograminearum*.

### 3.6. FpPer1 Involves in Resistance to Pesticide in F. pseudograminearum

**Figure 7:**
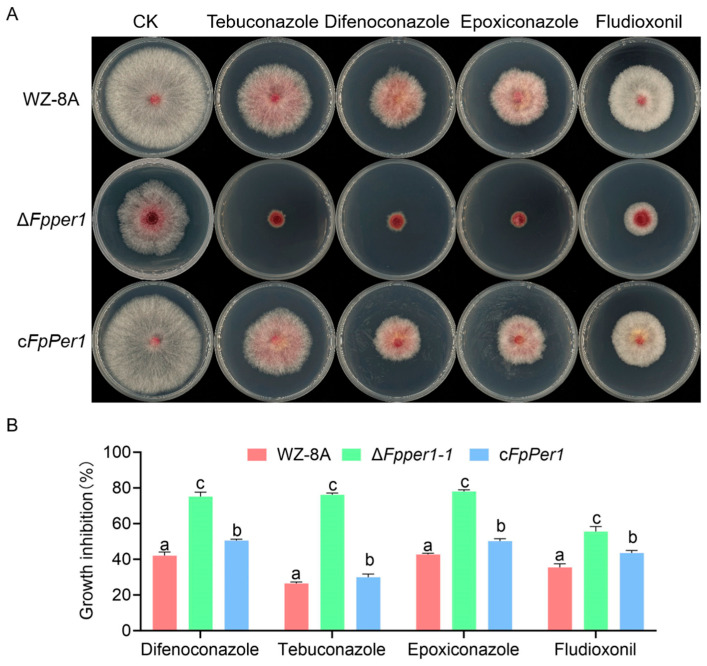
Sensitivity assays of WZ-8A, Δ*Fpper1-1*, and c*FpPer1* strains to various fungicides. (**A**), colonies of the strains grown on PDA medium with and without 0.084 ppm tebuconazole, 0.4 ppm difenoconazole, 0.1 ppm epoxiconazole, and 0.05 ppm fludioxonil. (**B**), colonies were photographed, and the inhibition rates were calculated for different fungicides. The mean values ± SD from three repetitions are presented. Lowercase letters (a–c) indicate significantly different groups as determined by Duncan’s multiple comparison procedures (*p* < 0.05).

Having established FpPer1’s involvement in tebuconazole resistance, we further investigated its potential role in conferring resistance to other fungicides. We selected three additional widely used fungicides that have demonstrated efficacy against *F. pseudograminearum*: difenoconazole, epoxiconazole, and fludioxonil. Interestingly, when the wild-type, Δ*Fpper1*, and complementary strain were cultured on PDA containing tebuconazole, difenoconazole, epoxiconazole, and fludioxonil, the mutant Δ*Fpper1-1* strain exhibits a stronger inhibition rate than that of the wild-type and complementary strain (Figure 7A,B). These consistent findings across multiple fungicide classes suggest that FpPer1 likely functions as a key regulator in a common resistance pathway targeted by diverse pesticides in *F. pseudograminearum*.

## 4. Discussion

Forward genetics, as a classical genetic approach that combines phenotypic screening with gene mapping, has been extensively validated as a powerful tool for identifying the functions of uncharacterized genes. In this study, we identified a GPI-anchored protein, FpPer1, involved in resistance to pesticides in *F. pseudograminearum* using a forward genetics method. Through integrated genetic and biochemical approaches, we demonstrated that FpPer1 played an important role in both pathogenicity and response to various environmental stresses. Our research on the *FpPER1* gene highlights its promise as a target for novel fungicides. Disrupting *FpPER1* could desynchronize fungal growth, reproduction, and infection processes, providing a precise, sustainable strategy for crop disease management.

Emerging evidence suggests that long-term use of single-action fungicides can induce the development of fungicide resistance for pathogens. The risk can be substantially mitigated through the combined application of fungicides with different mechanisms of action. For instance, combined application of fluazinam with triazole enhances the synergistic activity against *F. pseudograminearum* [22]. Similarly, it is an effective way to control fungal leaf diseases using the mixtures of flutriafol and azoxystrobin [23]. These findings highlight the importance of identifying novel fungicide targets in *F. pseudograminearum*. Previous reports indicated that Per1 modulates resistance to triazole drugs in *Aspergillus fumigatus* and *M. oryzae* [24,25]. We found that FpPer1 regulates resistance not only to triazole drug but also to fludioxonil, suggesting that FpPer1 may modulate multiple signaling pathways involved in pesticide resistance. Probably, the incompleteness of the cell wall protein in the Δ*Fpper1* mutant may be contribute to its increased sensitivity to pesticides. Another possible reason is that Afper1 supports cell development by modulating UPR-mediated ER homeostasis in *A. flavus* [26]. Disturbed ER homeostasis may also explain the loss of fungicide resistance in the Δ*Fpper1* mutant of *F. pseudograminearum.* Our findings reveal that FpPer1 plays a critical role in pesticide resistance in *F. pseudograminearum*. Furthermore, the Δ*Fpper1* mutant exhibited heightened sensitivity to antifungal agents, suggesting that FpPer1 is a potential molecular target for new fungicides. In the future, combining novel FpPer1-targeting compounds with triazole fungicides could significantly improve the management of *Fusarium* crown rot.

Disruption of *FpPER1* resulted in defects in both growth and pathogenicity in *F. pseudograminearum*, which may be attributed to defects in cellular integrity. Given that cell wall and membrane integrity are vital for maintaining cellular morphology, responding to environmental stress, and defending against pathogens [27,28], the observed hypersensitivity of the Δ*Fpper1* mutant to various environmental stresses suggests underlying issues with cellular integrity. Furthermore, pathogenic fungi depend on specialized infection structures, such as appressoria and penetration pegs, for successful host invasion [29,30,31,32]. While *MoPER1* has been demonstrated to regulate appressorium formation in *M. oryzae* [25], our findings reveal that the deletion of *FpPER1* impairs penetration pegs formation in *F. pseudograminearum*, representing a significant factor contributing to its reduced pathogenicity. In addition, GPI anchoring of protein is a conserved post-translational modification that occurs in endoplasmic reticulum [33]. However, it remains unclear whether the ER-localized protein FpPer1 regulates biological processes via lipid remodeling in *F. pseudograminearum*, which requires further investigation.

In conclusion, this study establishes FpPer1 as a multifunctional regulator of pathogenicity and pesticide resistance in *F. pseudograminearum*. Our findings provide a theoretical basis for the development of new fungicides targeting the gene *FpPER1* and offer novel insights into adaptation mechanisms. Future research should focus on elucidating the molecular networks through which FpPer1 coordinates these diverse biological processes.

## Data Availability

The original contributions presented in this study are included in the article and Supplementary Material. Further inquiries can be directed to the corresponding authors.

## Supplementary Materials

The following supporting information can be downloaded at: https://www.mdpi.com/article/10.3390/jof11090673/s1, Figure S1: The sequences identified using nested-PCR; Table S1: The primers used in this study.
