# Supplementary material for: T-DNA Mutagenesis Reveals FpPer1 as a Dual-Function Regulator of Virulence and Fungicide Resistance in Fusarium pseudograminearum
Source: J Fungi (Basel). 2025 Sep 12;11(9):673. doi: 10.3390/jof11090673 (PMC12470859; doi:10.3390/jof11090673)
Supplement: Supplementary file 1 [file jof-11-00673-s001.zip › jof-3848950-supplementary.pdf]

Supplementary Materials:

TAGCAAAGCTGCTCTAGCATTCGCCCTTCCGCAGTTGCGCAGGTGTTTAGCCAAACAGGTATTTCGAG
AACGCCGACGTTACGTCGGGTTGCCAATGAGCCACATCGTCAACTGGTCTCTCCTCTCTCATGCAGTT
TGACTTTTAATTATCCAACTTCTTTTTTTCCCCTTCGCAAACAGGTCGATGCACTCGCAATGGCAACTC
AAAGGGGGCGGCCATGGGCGCGGATTATATCCTTGACCGTCCTCGTTCTCGCTCTTGCGATTACCGTT
GATGCTTCAACTGGCGACAGGTTGCCGGAATTCAAGGACTGTCTCAAGGTAAGGGTAACCACAGCGC
AAAGTGGCCAGAGACTGATTCATATTTATAGATCTGCAATGCCGAGAATTGCGCACCAAACAAACCG
CAAACTCCTATCCGTACGCGTCCTTGATTCTGCCATCAGAGTGCTACAAACACTGATAGTTTAAACTG
AAGGCGGGAAACGACAATCTGATCCAAGCTCAAGCTGCTATAGCATTCGCCACTTGGGCTGGGGCAA
CTGTTGGGAAGGGGCCAATGCGAGAGCAGCTTGCGCT

Figure S1. the sequence identified using nested-PCR.

Table S1. The primers used in this study.

Primers	Sequences (5'-3')	Function
Per1- 1F	CGGTCTGGCGAAAGGGTTA	Amplification of upstream and downstream homologous arm fragments of the *FpPER1* gene
Per1-2R	TTGACCTCCACTAGCTCCAGCCAAGCCTGCG AGTGCATCGACCTGTT	
Per1- 3F	ATAGAGTAGATGCCGACCGCGGGTTCTAATGA GCAGACTCT	
Per1-4R	TCAGGTATGCCACTATTGAGCC	
Per1- 5F	GCTTGGCAAACGAATAGGTAGT	Screening of *FpPER1* gene knockout transformants and complemented strain
Per1- 6R	TGTCACATCGCCTGCTCTA	
NF	GCTCTTGCGATTACCGTTGATG	
NR	ATCCAAGCAACGACGAAACCAG	
HYG/F	GGCTTGGCTGGAGCTAGTGGAGGTCAA	
HY/R	GTATTGACCGATTCCTTGCGGTCCGAA	
YG/F	GATGTAGGAGGGCGTGGATATGTCCT	
HYG/R	GAACCCGCGGTCGGCATCTACTCTAT	
H852	AACTCACCGCGACGTCTGTC	
H850	TTGTCCGTCAGGACATTGTT	
H855R	GCTGATCTGACCAGTTGC	
H856F	GTCGATGCGACGCAATCGT	
